# Proteomic Analysis of Atrial Appendages Revealed the Pathophysiological Changes of Atrial Fibrillation

**DOI:** 10.3389/fphys.2020.573433

**Published:** 2020-09-16

**Authors:** Ban Liu, Xiang Li, Cuimei Zhao, Yuliang Wang, Mengwei Lv, Xin Shi, Chunyan Han, Pratik Pandey, Chunhua Qian, Changfa Guo, Yangyang Zhang

**Affiliations:** ^1^Department of Cardiology, Shanghai Tenth People’s Hospital, Tongji University School of Medicine, Shanghai, China; ^2^Department of Cardiology, The First Affiliated Hospital of Chongqing Medical University, Chongqing Medical University, Chongqing, China; ^3^Department of Cardiology, Tongji Hospital, Tongji University School of Medicine, Shanghai, China; ^4^Department of Immunology, School of Basic Medical Science, Nanjing Medical University, Nanjing, China; ^5^Shanghai East Hospital of Clinical Medical College, Nanjing Medical University, Shanghai, China; ^6^Department of Cardiovascular Surgery, Shanghai East Hospital, Tongji University School of Medicine, Shanghai, China; ^7^Department of Pediatric Cardiology, Xinhua Hospital, School of Medicine, Shanghai Jiao Tong University, Shanghai, China; ^8^Department of Endocrinology and Metabolism, Shanghai Tenth People’s Hospital, Tongji University School of Medicine, Shanghai, China; ^9^Department of Cardiovascular Surgery, Zhongshan Hospital, Fudan University, Shanghai, China

**Keywords:** atrial fibrillation, proteomics, proteins, structural remodeling, mechanism

## Abstract

Atrial fibrillation (AF), known as the most common arrhythmia in the developed world, affects 1.5–2.0% of the population. Numerous basic studies have been carried out to identify the roles of electric and structural remodeling in the pathophysiological changes of AF, but more explorations are required to further understand the mechanisms of AF development. Proteomics enables researchers to identify protein alterations responsible for the pathological developing progresses of diseases. Compared to the genome, the proteome is closely related to the disease phenotype and can better manifest the progression of diseases. In this study, AF patients proteomically analyzed to identify possible mechanisms. Totally 20 patients undergoing cardiac surgery (10 with paroxysmal AF and 10 with persistent AF) and 10 healthy subjects were recruited. The differentially expressed proteins identified here included AKR1A1, LYZ, H2AFY, DDAH1, FGA, FGB, LAMB1, LAMC1, MYL2, MYBPC3, MYL5, MYH10, HNRNPU, DKK3, COPS7A, YWHAQ, and PAICS. These proteins were mainly involved in the development of structural remodeling. The differently expressed proteins may provide a new perspective for the pathological process of AF, and may enable useful targets for drug interference. Nevertheless, more research in terms of multi-omics is required to investigate possible implicated molecular pathways of AF development.

## Introduction

Atrial fibrillation (AF), known as the most common arrhythmia in the developed world, attacks 1.5–2.0% of the population. In the population aged over 40 years, the lifetime risk for AF is about 25% both in genders (Heeringa et al., 2006). The incidence of AF has risen about threefold with the aging population during the next 50 years, which progressively increases economic burden (Steinberg, 2004; Miyasaka et al., 2006). AF is characterized electrocardiographically by low-amplitude baseline oscillations as supraventricular arrhythmia. The fibrillatory waves, namely f waves, originate from the fibrillating atria and are accompanied by an irregular ventricular rhythm. AF mainly causes cardiovascular mortality and morbidity (Heeringa et al., 2006). A variety of cardiac diseases and conditions may cause atrial remodeling and consequently lead to AF development, but AF may also contribute to atrial remodeling owing to the progressiveness of the arrhythmia (Wakili et al., 2011).

These remodeling approaches include structural remodeling characterized as atrial fibrosis (Frustaci et al., 1997) and atrial adipose (Hatem and Sanders, 2014), electrical remodeling featured by changes in ion channels and gap junction proteins (Lai et al., 1999), and endocardial and metabolic remodeling (Schild et al., 2006; Jeganathan et al., 2017). Numerous basic studies have been conducted to explore the roles of electric, structural and contractile remodeling in the pathophysiological changes of AF. Nevertheless, further explorations are required to better understand the mechanisms of AF development.

Various techniques, especially “omics” techniques, have been applied to identify the molecular targets and mechanisms that mediate AF-related remodeling. Proteomics is one “omics” technique to study large-scale gene expression at the protein level, and enables researchers to identify protein alterations responsible for the pathological developing progresses of diseases. The proteome determines the cell phenotype and variations that may change cell and tissue functions. Compared to the genome, the proteome is closely related to the disease phenotype and can better manifest the progression of diseases.

In this study, AF patients were categorized into two groups according to the duration of AF. Paroxysmal AF was termed as terminating spontaneously within 7 days, and permanent AF was defined as persisting for more than 1 year. We compared the proteomics between subjects with sinus rhythm (SR) and patients with AF to demonstrate the pathophysiological changes.

## Materials and Methods

### Patients and Tissue Preparation

Thirty subjects were enrolled and divided into three groups, including 10 healthy subjects with SR (Group1, G1), 10 patients with paroxysmal AF (Group2, G2), and 10 patients with permanent AF (Group3, G3). The 10 healthy subjects with SR were all males and aged between 25 and 38 years old. All AF patients were subjected to physical examination and clinical evaluation, including medical history, routine blood test, electrocardiography (ECG), chest CT, and echocardiography. Exclusion criteria were valvular heart disease, coronary artery disease, chronic heart failure, myocarditis, cardiomyopathy, chronic pulmonary heart disease, or hyperthyroidism.

Protocol for sample collection was adhered to the Human Ethics Committee of Shanghai East Hospital (DI:0402017). This study complied with the Helsinki Declaration. Prior to operation of fibrillation ablation, written informed consents were obtained from all enrolled patients. The left atrial appendage (LAA) was resected during isolated surgical ablation, and tissue samples were collected from the abandoned LAA. Normal LAA samples were collected from healthy male donors. Collected tissues were frozen in −80°C liquid nitrogen before further processing.

### Protein Extraction

The extraction of proteins from atrial tissues followed previous protocols (Waller et al., 2013). Briefly, about 20 mg of atrial tissues were cut on ice and homogenized in a buffer, containing 100 mM Tris, 4% SDS, and maintaining PH 7.6. Protease and phosphatase inhibitors from Meck were added in the buffer. The mixture was sonicated for 5 s at 15% amplitude on ice and paused for 5 s for 2 min of working time on a JY92-IIDN instrument (Ningbo Scientz Biotechnology Co., Ltd., China). The proteins were denatured and condensed for 5 min at 95°C circumstance afterword. The mixture was centrifuged at 14,000 g for 10 min to remove the insoluble debris and retain the supernatant for proteomic experiments. The bicinchoninic acid (BCA) assay was performed to determine the concentration of protein. All protein samples were stored at −80°C for further experiment.

### Label-Free Proteomic Analysis

Protein digestion was performed by Filter-aided sample preparation (FASP) (Wisniewski et al., 2009). Briefly, protein extraction 200 μg was mixed with a reducing buffer (1 M DTT) to 100 mM DTT concentration as total, incubated for 1 h at 56°C afterword. Then the protein samples were washed twice with 200 mL of a UA buffer (pH 8.5, 8 Murea in 0.1 M Tris-HCl), adding 50 mM iodoacetamide in the tube to alkylate in the darkness for 30 min. The mixture was washed firstly with the 100 mL UA buffer and secondly with ammonium bicarbonate 50 mM for three times. All resulting solutions were centrifuged at 25°C for 12,000 g. Protein samples were digested with trypsin (Promega) at 37°C for 18 hr, with a 1:50 (w/w) concentration in 50 mM ammonium bicarbonate. Then, peptide samples were centrifuged to elute. The BCA protein procedure was used to determine peptide concentration. Peptides were desalted and dried for further procedure.

For proteomic analysis, nanoflow HPLC Easy-nLC 1000 system (Thermo Fisher Scientific) was used to separate about 1 μg peptides at 300 nL/min with a 70-min LC gradient. Proteomic analyses were conducted on an Orbitrap Fusion mass spectrometer (Thermo Fisher Scientific). The positive ion mode at 1,900 V was set as spray voltage and the ion transfer tube at 275°C was also set. Xcalibur was used to perform data-dependent acquisition. The orbitrap mass analyzer, with a RF lens 60%, resolution of 60,000 @ m/z 200, maximum IT 50 ms and AGC target 4e5, was used to perform the MS1 full scan. HCD fragmentation, with a resolution of 15,000 @m/z 200, maximum IT 150 ms in a 3 s cycle time and AGC target 2e5, was used to generate top-speed MS2 scans. 1.2 m/z was set as isolation window. The HCD collision energy and the dynamic exclusion time were set at 30% and 60 s separately. MS2 analysis were selected by precursors charged at state 2–6.

### Database for Proteomic Analysis

MaxQuant 1.6.1.0, containing 172,418 sequences (downloaded in July, 2019), was used to analyze all mass spectra. Enzyme specification was used in search. The fixed modification was performed as carbamidomethylation of cysteine, while variable modification was carried out by N-terminal acetylation and oxidation of methionine. In the initial scan and the main search setting at 6 ppm, mass tolerances for fragment ions and precursor were set at 0.02 Da and 20 ppm respectively.

The Andromeda search engine, integrating into Maxquant, was used to search tandem MS. Seven amino acids was set as cutoff of minimum peptide length, while two amino acids was set as maximum permissible missed cleavage. Maximal FDR was set at 0.01for proteins, peptide spectral match and site. Two sequence-unique peptides was set as minimum identification.

The label-free quantitation (LFQ) was analyzed by the Andromeda search engine. The quantification results of Maxquant protein and peptide were imported for further analysis. Comparing with controls, differentially expressed proteins in patients were defined as significant change if the ratios were ≥2 or ≤0.5 (*P* < 0.05).

### Protein-Protein Interaction (PPI) Network Analysis

Proteins and their interactive functions form the backbone of cellular biology. The PPIs were identified and characterized to necessarily understand the physiology and efficacy in the organism. The connective network was demonstrated for full understanding of cellular machinery. STRING 11.0 (Szklarczyk et al., 2019)^1^ covering more than 5,090 organisms was used to analyze PPIs. The biological characteristics of high-throughput transcriptome data was identified by Gene ontology (GO) analysis in defining protein products. GO^2^ consortium was used to identify the pathways involved. For molecular function in terms of GO analysis, *p* <0.05 was considered significant.

## Results

### Patient Characteristics

The baseline characteristics of AF patients, with paroxysmal or permanent AF, were shown in Table 1. All AF patients received transthoracic echocardiography to rule out heart failure, defined as left ventricular ejection fraction or LVEF ≥50%, and valvular heart disease. Color Doppler echocardiography measured left atrial diameter before fibrillation ablation. All subjects went through coronary CT angiography (CCTA) to rule out coronary heart disease.

**TABLE 1:** Baseline Characteristics of patients with paroxysmal or permanent AF.

No.	Type of AF	Gender	Age (Year)	Height (M)	Weight (Kg)	Hyper lipidemia	Smoking	Hyper tension	T2DM	LVEF (%)	CCTA	LAD (mm)	Duration of AF (Year)
1	Paroxysmal	Male	69	1.69	76	No	No	Yes	No	70	Negative	40	/
2	Paroxysmal	Male	63	1.7	64	No	No	No	No	59	Negative	46	/
3	Paroxysmal	Male	63	1.7	70	No	No	No	No	66	Negative	39	/
4	Paroxysmal	Male	69	1.73	67	No	No	Yes	No	67	Negative	46	/
5	Paroxysmal	Male	69	1.65	75	No	No	No	No	70	Negative	36	/
6	Paroxysmal	Male	61	1.76	76	No	No	Yes	Yes	60	Negative	42	/
7	Paroxysmal	Male	64	1.68	52	No	Yes	No	No	64	Negative	40	/
8	Paroxysmal	Male	64	1.81	71	No	No	Yes	Yes	63	Negative	39	/
9	Paroxysmal	Male	61	1.67	87	No	Yes	Yes	No	62	Negative	37	/
10	Paroxysmal	Male	66	1.73	82	No	No	Yes	No	63	Negative	42	/
11	Persistent	Male	63	1.76	86	No	No	Yes	No	57	Negative	46	2.5
12	Persistent	Male	63	1.78	80	No	No	No	No	68	Negative	55	3
13	Persistent	Male	64	1.7	70	No	No	No	No	67	Negative	41	4
14	Persistent	Male	64	1.64	84	No	No	Yes	No	55	Negative	48	2
15	Persistent	Male	65	1.69	73	No	No	Yes	No	69	Negative	55	3.5
16	Persistent	Male	66	1.68	66	No	No	Yes	No	64	Negative	45	4
17	Persistent	Male	67	1.75	80	No	No	Yes	Yes	59	Negative	47	2.5
18	Persistent	Male	67	1.65	73	No	Yes	Yes	No	59	Negative	47	3
19	Persistent	Male	63	1.64	61	No	No	No	No	73	Negative	49	2
20	Persistent	Male	67	1.78	90	No	No	Yes	No	70	Negative	58	2.5

*AF, Atrial fibrillation; T2DM, type 2 diabetes mellitus; LVFE, left ventricular ejection fraction; LAD, Left atrial diameter.*

### Differentially Expression of Proteins

**FIGURE 1:**
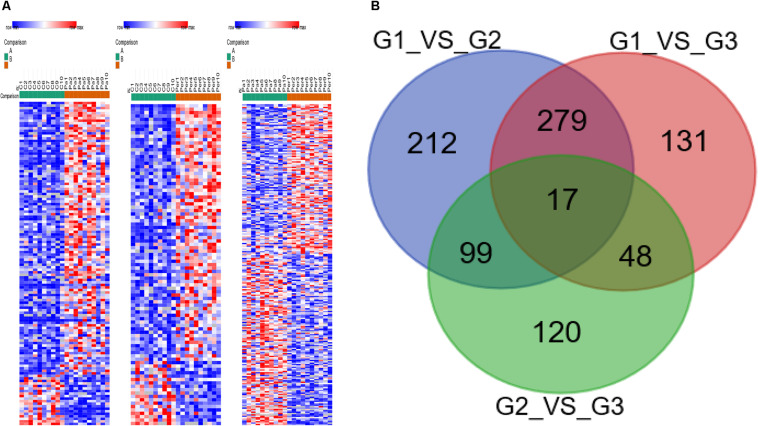
Heatmap visualization of the differently expressed proteins identified in healthy controls, paroxysmal AF patients, and permanent AF patients. **(A)** Heatmap showing the differential protein expression profiles compared in pairs among three groups. **(B)** The differently expressed proteins identified in three groups by Venn diagram via comparing in pairs.

Three groups of specimens were detected by liquid chromatography-tandem mass spectrometry (LC-MS/MS) and analyzed by the LFQ proteomics. This method quantified 3,911 proteins. The proteome of LAAs was examined to compare the different changes in protein expressions between healthy controls and AF patients, using LFQ intensities. The differentially expressed proteins, compared in pairs between three groups, were shown in the heat map (Figure 1A). Totally 17 differentially expressed proteins with significant difference were identified with a gradient change among healthy controls, paroxysmal AF group, and permanent AF group via comparing in pairs (Figure 1B).

### Functions of the Identified Proteins

The 17 proteins were divided into three major groups according to their different functions: association with cytoskeleton and protein binding, with chromatin binding, and with oxidative stress (Table 2).

**TABLE 2:** Differently expressed proteins identified by proteomic analysis.

No.	Protein name	Gene	Accession no.	Function
1	Epididymis secretory protein Li 6	AKR1A1	V9HWI0	Cardiac necrosis
2	Lysozyme	LYZ	B2R4C5	Regulating apoptosis and K(ATP) ion channel
3	Core histone macro-H2A.1	H2AFY	O75367	Promoter-specific chromatin binding, oxidative stress
4	Dimethylargininedimethy laminohydrolase 1	DDAH1	B1AKK2	Sarcolemma of cardiomyocytes
5	Fibrinogen alpha chain	FGA	P02671	Structural molecule activity, metabolism
6	Epididymis secretory sperm binding protein Li 78p	FGB	V9HVY1	Structural molecule activity
7	Laminin subunit beta-1	LAMB1	P07942	Structural molecule activity
8	Laminin gamma 1	LAMC1	A0A024R972	Structural molecule activity
9	MYL2 protein	MYL2	Q6IB42	Structural molecule activity
10	Mutant cardiac myosin-binding protein C	MYBPC3	B6D425	Structural molecule activity
11	Myosin light chain 5	MYL5	D6RA88	Structural molecule activity
12	Myosin-10	MYH10	P35580	Actin binding
13	Heterogeneous nuclear ribonucleoprotein U	HNRNPU	Q00839	Actin binding
14	Dickkopf-related protein 3	DKK3	Q9UBP4	Cardiac hypertrophy and fibrosis
15	COP9 signalosome complex subunit 7a	COPS7A	Q9UBW8	Cytosol of cardiomyocytes, cardiac proteinopathy
16	14-3-3 protein theta	YWHAQ	P27348	Adaptor protein, regulating electric channel activity
17	Multifunctional protein ADE2	PAICS	E9PBS1	Purine biosynthesis, unclear

### PPI Network

**FIGURE 2:**
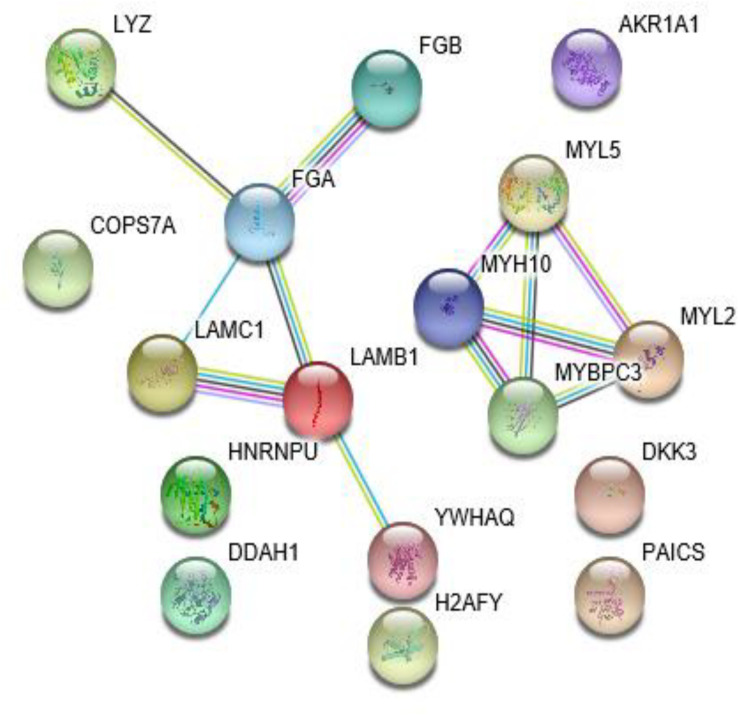
The protein-protein interaction network for the 17 identified proteins.

The STRING analysis was used to establish a PPI network involving the 17 differentially expressed proteins (Figure 2). This network contained 17 nodes and 12 edges. The average node degree was 1.41. In the network analysis, the clustering coefficient (cc) was 0.5, and PPI enrichment *p*-value was 4.94e-05, which was practically negligible.

**FIGURE 3:**
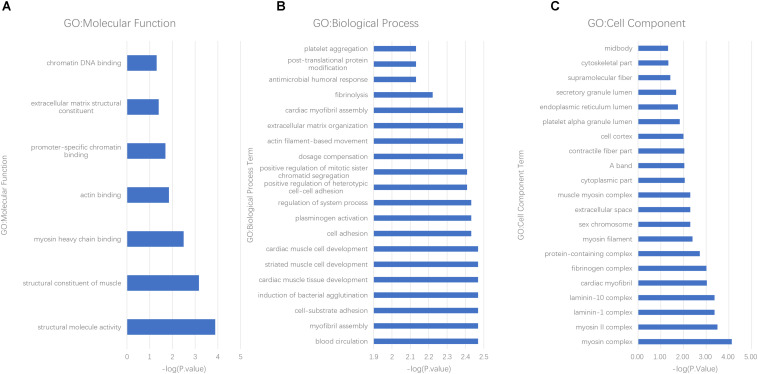
GO analysis of differently expressed proteins in AF patients. **(A)** molecular function, **(B)** biological process, and **(C)** cell components.

Molecular function (MF), cellular component (CC), and biological process (BP) were all analyzed by the GO consortium database. MF analysis suggested that most of the differently expressed proteins participated in structural component, protein binding, and chromatin DNA binding (Figure 3A). BP analysis demonstrated these proteins were mostly involved in myocyte activity, development, metabolism, post-translational protein modification, cell-substrate interaction, and apoptotic regulation (Figure 3B). CC analysis showed cellular structural components (Figure 3C).

## Discussion

AF is the major cause of thrombotic stroke (Vergara and Della Bella, 2014). Though AF is a major cause of mortality and morbidity and there are decades of basic and clinical studies, its fundamental mechanisms and effective treatment are still unknown. Patients with paroxysmal AF suffer less than 7 days of self-terminating episodes, but mostly progress to persistent AF, lasting more than 7 days (Kerr et al., 2005). AF lasting over 12 months is termed “long-term persistent AF” or permanent AF. AF leads to structural and electrical remodeling of the atria, while the underlying mechanisms are scarcely acquainted and remain to be revealed. Proteins are essential in cellular function and biological component, and make up to about 50% of the structural component of mammalian cells (Milo, 2013). The proteome represents the entire set of proteins expressed based on cellular genome at a specific time point, while various cellular processes and disease developments are always manifested with different protein levels (Mann et al., 2013). In brief, characterizing proteomes and specific proteins have almost been a new approach to understand the cell function mechanism and disease development.

In this study, 30 LAA samples underwent proteomic analysis, and 17 differently expressed proteins were identified between healthy subjects and patients with AF after compared in pairs between three groups, which means gradient changes with the development of AF. With time progressing, the atrial remodeling continuously occurs, and paroxysmal AF evolves into permanent AF (Jalife and Kaur, 2015). All these differently expressed proteins were grouped according to their functions in AF development and progression, including proteins associated with apoptosis, with cytoskeleton and protein binding, with oxidative stress, and with ion channel regulation.

### Cardiomyocytes Necrosis and Apoptosis

AKR1A1 belongs to the aldo/keto reductase superfamily, consisting of more than 40 known proteins and enzymes. This superfamily is also involved in the reduction of xenobiotic and biogenic aldehydes, virtually presenting every tissue, and is known as aldehyde reductase. AKR1A1 protein levels increased in cardiac tissues with more vacuole formation and severe necrosis. These results suggest that AKR1A1 protein participates in DOX-induced cardiotoxicity (Zhou et al., 2016). LYZ levels were elevated in cardiac sarcoidosis patients with intractable heart failure and refractory arrhythmias (Odawara et al., 2019). LYZ may participate in the apoptosis in the isolated hearts of rats (Kim et al., 2010).

### Oxidative Stress

H2AFY, belonging to histone H2A family, supersedes conventional H2A histones with a subset of nucleosomes. Histones, basic nuclear proteins in eukaryotes, constitute the nucleosome structure of the chromosomal fiber. Human zinc finger RNA-binding protein is regulated in macrophage differentiation by preventing aberrant splicing of H2AFY, and controls interferon signaling. H2AFY may participate in transcriptional response to infection (Haque et al., 2018). H2AFY is related to inflammation in healthy subjects exposed to ultrafine carbon particles, and especially changes in the glucose metabolism and cardiovascular system (Huang et al., 2010).

### Cytoskeleton and Protein Binding

Twelve proteins differently expressed between healthy controls and AF patients, correlating to cytoskeletal structure, were identified. DDAH1 attenuates ventricular remodeling and cardiac hypertrophy under stress conditions via regulating subcellular NO signaling (Xu et al., 2017). FGA participates in left ventricular diastolic dysfunction as a core protein in β3-adrenergic receptor knockout mice. FGA may potentially relate to the cardiac muscle contraction and actin cytoskeleton organization (Yang et al., 2019). FGB mutation can elevate the level of plasma fibrinogen in AF patients, and thereby played a role in cardioembolic stroke (Hu et al., 2017).

LAMB1 and LAMC1 belonging to an extracellular matrix glycoprotein family constitute non-collagenous basement membranes. LAMB1 is moderately expressed in heart basement membranes (Cotrufo et al., 2005). Coding exons of LAMB1, LAMB4 and PIK3CG were screened in dilated cardiomyopathy (Schonberger et al., 2005). LAMC1-deficient cardiomyocytes lacked basement membranes, leading to hormonal regulation and electrical activity (Malan et al., 2009).

MYL2 triggers contraction by phosphorylation of the regulatory light chain. Mutations in this gene are related to hypertrophic cardiomyopathy. MYBPC3, a myosin- associated protein, consists in the cross-bridge-bearing zone of A bands in striated muscles, and is expressed exclusively in heart muscles. Genetic testing discovered the prevalence of MYBPC3 and MYL2in patients with hypertrophic cardiomyopathy and AF (Bongini et al., 2016). MYL5 is a component of the hexameric ATPase cellular motor protein myosin. MYH10, belonging to the superfamily of myosins, is a conventional non-muscle myosin. MYH10 is an actin-dependent motor protein, regulating cytokinesis, cell polarity, and cell motility. Mutations in MYH10 are associated with cardiac developmental defects (Takeda et al., 2003; Lo et al., 2004).

HNRNPU, belonging to a protein superfamily, binds nucleic acids and functions in the nucleus by the formation of ribonucleoprotein complexes with heterogeneous nuclear RNA. Mice lacking HNRNPU developed lethal dilated cardiomyopathy, which presented disorganized cardiomyocytes, abnormal excitation-contraction coupling activities, and impaired contractility (Ye et al., 2015). DKK3, belonging to the Dickkopf family as a secreted protein, plays an important role in heart development. DKK3 presents cardioprotective effect in pathological cardiac hypertrophy via regulating the ASK1-JNK/p38 signaling pathway (Zhang et al., 2014). COPS7A, a component of the COP9 signalosome, may participate in regulating the degradation of a bona fide misfolded and a surrogate protein in the myocardial cytosol, while COPS8 hypomorphism may impair autophagosome and exacerbate cardiac proteinopathy (Liu et al., 2016).

Besides the structural functions above, YWHAQ was suggested to amplify and prolong the activity of beta-adrenergic stimulated HERG channel by affecting IKr activity in ventricular repolarization (Choe et al., 2006). LYZ may participate in K (ATP) ion channel activity in the isolated hearts of rats, in addition to apoptosis of cardiomyocyte (Kim et al., 2010).

Besides all the 16 differently expressed proteins discussed above, the cardiac function of PAICS, which may participate in purine biosynthesis, is unclear.

AF is commonly associated with structural and electrical atrial remodeling. Structural atrial remodeling mainly includes degenerative processes, such as apoptosis and fibrosis, and alteration of cellular structural expression. Oxidative stress acts as an interdependent signaling pathway leading to cardiac fibrosis (Schotten et al., 2011).

From the above analysis, we speculate that most differently expressed proteins participate in structural remodeling and some may further develop to atrial electrical remodeling by structural remodeling or direct electrophysiologic consequence.

In this study, label-free proteomics analysis identified 17 AF-associated proteins, which were mostly correlated to structural atrial remodeling. It has been well-demonstrated for decades that AF represents atrial myocardium hypertrophy, atrial cavity dilatation, and apoptosis of atrial cardiomyocytes, and replaces with fibrotic tissue focus or diffusion. Whether the development of arrhythmia precedes or follows the structural remodeling is unclear. Underlying this sophisticated multiple process is a complex network of molecular correlation. In the study, a comprehensive PPI network was generated from the proteomics approach to define the molecular functions participated in AF development. As mentioned above, these proteins were structural components of cardiomyocyte, and structural remodeling was presumed to play a crucial part in AF development.

The differentially expressed proteins in AF patients require further investigation to understand their exact roles in the pathological process of AF. For further functional research, candidate proteins will be selected by stringent bioinformatics analysis, which may provide vital information to investigators for future research. The joint analysis of multi-omics analysis will be carried out to reveal the regulatory mechanism of AF.

### Limitations

The sample size of investigated subjects was small, owing to the difficulty in obtaining LAA samples. The healthy controls were younger than AF patients on average, which may result in inconsistency of the samples. In addition, we investigated human samples with idiopathic disease, but experiments were hardly carried out to modulate the protein levels. Although the left atrium is the key player in AF, only left atrial appendage tissues can be resected during cardiac ablation, which cannot fully represent the pathological changes of AF in the left atrium and cannot thoroughly explain the mechanism of AF.

## Data Availability Statement

The raw data supporting the conclusions of this article will be made available by the authors, without undue reservation, to any qualified researcher.

## Ethics Statement

The studies involving human participants were reviewed and approved by the Ethics Committee of the Shanghai East Hospital. The patients/participants provided their written informed consent to participate in this study.

## Author Contributions

YZ, CG, and CQ conceived and designed the experiments. BL, YW, and ML performed the experiments. XS, CH, and PP analyzed the data. BL, XL, and CZ wrote the manuscript. All authors contributed to the article and approved the submitted version.

## Conflict of Interest

The authors declare that the research was conducted in the absence of any commercial or financial relationships that could be construed as a potential conflict of interest.

## Footnotes

1 https://string-db.org/

2 http://www.geneontology.org/

## References

[B1] Bongini C. Ferrantini C. Girolami F. Coppini R. Arretini A. Targetti M. (2016). Impact of genotype on the occurrence of atrial fibrillation in patients with hypertrophic cardiomyopathy. *Am. J. Cardiol.* 117 1151–1159. 10.1016/j.amjcard.2015.12.058 26869393

[B2] Choe C. U. Schulze-Bahr E. Neu A. Xu J. Zhu Z. I. Sauter K. (2006). C-terminal HERG (LQT2) mutations disrupt IKr channel regulation through 14-3-3epsilon. *Hum. Mol. Genet.* 15 2888–2902. 10.1093/hmg/ddl230 16923798

[B3] Cotrufo M. De Santo L. Della Corte A. Di Meglio F. Guerra G. Quarto C. (2005). Basal lamina structural alterations in human asymmetric aneurismatic aorta. *Eur. J. Histochem. [EJH]* 49 363–370. 10.4081/964 16377578

[B4] Frustaci A. Chimenti C. Bellocci F. Morgante E. Russo M. A. Maseri A. (1997). Histological substrate of atrial biopsies in patients with lone atrial fibrillation. *Circulation* 96 1180–1184. 10.1161/01.cir.96.4.1180 9286947

[B5] Haque N. Ouda R. Chen C. Ozato K. Hogg J. R. (2018). ZFR coordinates crosstalk between RNA decay and transcription in innate immunity. *Nat. Commun.* 9:1145. 10.1038/s41467-018-03326-5 PMC5861047 29559679

[B6] Hatem S. N. Sanders P. (2014). Epicardial adipose tissue and atrial fibrillation. *Cardiovasc. Res.* 102 205–213. 10.1093/cvr/cvu045 24648445

[B7] Heeringa J. van der Kuip D. A. Hofman A. Kors J. A. van Herpen G. Stricker B. H. (2006). Prevalence, incidence and lifetime risk of atrial fibrillation: the rotterdam study. *Eur. Heart J.* 27 949–953. 10.1093/eurheartj/ehi825 16527828

[B8] Hu X. Wang J. Li Y. Wu J. Qiao S. Xu S. (2017). The beta-fibrinogen gene 455G/A polymorphism associated with cardioembolic stroke in atrial fibrillation with low CHA2DS2-VaSc score. *Sci. Rep.* 7:17517. 10.1038/s41598-017-17537-1 PMC5727505 29235504

[B9] Huang Y. C. Schmitt M. Yang Z. Que L. G. Stewart J. C. Frampton M. W. (2010). Gene expression profile in circulating mononuclear cells after exposure to ultrafine carbon particles. *Inhalat. Toxicol.* 22 835–846. 10.3109/08958378.2010.486419 20507211 PMC3855293

[B10] Jalife J. Kaur K. (2015). Atrial remodeling, fibrosis, and atrial fibrillation. *Trends Cardiovas. Med.* 25 475–484. 10.1016/j.tcm.2014.12.015 25661032 PMC5658790

[B11] Jeganathan J. Saraf R. Mahmood F. Pal A. Bhasin M. K. Huang T. (2017). Mitochondrial dysfunction in atrial tissue of patients developing postoperative atrial fibrillation. *Ann. Thorac. Surg.* 104 1547–1555. 10.1016/j.athoracsur.2017.04.060 28760472

[B12] Kerr C. R. Humphries K. H. Talajic M. Klein G. J. Connolly S. J. Green M. (2005). Progression to chronic atrial fibrillation after the initial diagnosis of paroxysmal atrial fibrillation: results from the Canadian registry of atrial fibrillation. *Am. Heart J.* 149 489–496. 10.1016/j.ahj.2004.09.053 15864238

[B13] Kim Y. J. Lim H. J. Choi S. U. (2010). Effect of propofol on cardiac function and gene expression after ischemic-reperfusion in isolated rat heart. *Korean J. Anesthesiol.* 58 153–161. 10.4097/kjae.2010.58.2.153 20498794 PMC2872860

[B14] Lai L. P. Su M. J. Lin J. L. Lin F. Y. Tsai C. H. Chen Y. S. (1999). Down-regulation of L-type calcium channel and sarcoplasmic reticular Ca(2+)-ATPase mRNA in human atrial fibrillation without significant change in the mRNA of ryanodine receptor, calsequestrin and phospholamban: an insight into the mechanism of atrial electrical remodeling. *J. Am. CollCardiol.* 33 1231–1237. 10.1016/s0735-1097(99)00008-x 10193721

[B15] Liu J. Su H. Wang X. (2016). The COP9 signalosome coerces autophagy and the ubiquitin-proteasome system to police the heart. *Autophagy* 12 601–602. 10.1080/15548627.2015.1136773 26760900 PMC4836033

[B16] Lo C. M. Buxton D. B. Chua G. C. Dembo M. Adelstein R. S. Wang Y. L. (2004). Nonmuscle myosin IIb is involved in the guidance of fibroblast migration. *Mol. Biol. Cell* 15 982–989. 14699073 10.1091/mbc.E03-06-0359 PMC363055

[B17] Malan D. Reppel M. Dobrowolski R. Roell W. Smyth N. Hescheler J. (2009). Lack of laminin gamma1 in embryonic stem cell-derived cardiomyocytes causes inhomogeneous electrical spreading despite intact differentiation and function. *Stem Cells (Dayton, Ohio)* 27 88–99. 10.1634/stemcells.2008-0335 18927478

[B18] Mann M. Kulak N. A. Nagaraj N. Cox J. (2013). The coming age of complete, accurate, and ubiquitous proteomes. *Mol. Cell* 49 583–590. 10.1016/j.molcel.2013.01.029 23438854

[B19] Milo R. (2013). What is the total number of protein molecules per cell volume? A call to rethink some published values. *BioEssays* 35 1050–1055. 10.1002/bies.201300066 24114984 PMC3910158

[B20] Miyasaka Y. Barnes M. E. Gersh B. J. Cha S. S. Bailey K. R. Abhayaratna W. P. (2006). Secular trends in incidence of atrial fibrillation in olmsted county, minnesota, 1980 to 2000, and implications on the projections for future prevalence. *Circulation* 114 119–125. 10.1161/circulationaha.105.595140 16818816

[B21] Odawara K. Inoue T. Hirooka Y. (2019). Effective steroid therapy in an elderly patient with cardiac sarcoidosis and severe left ventricular dysfunction. *J. Cardiol. Cases* 19 165–168. 10.1016/j.jccase.2018.12.019 31073350 PMC6495045

[B22] Schild L. Bukowska A. Gardemann A. Polczyk P. Keilhoff G. Täger M. (2006). Rapid pacing of embryoid bodies impairs mitochondrial ATP synthesis by a calcium-dependent mechanism–a model of in vitro differentiated cardiomyocytes to study molecular effects of tachycardia. *Biochimica et Biophysicaacta* 1762 608–615. 10.1016/j.bbadis.2006.03.005 16644187 PMC3153943

[B23] Schonberger J. Kuhler L. Martins E. Lindner T. H. Silva-Cardoso J. Zimmer M. (2005). A novel locus for autosomal-dominant dilated cardiomyopathy maps to chromosome 7q22.3-31.1. *Hum. Genet.* 118 451–457. 10.1007/s00439-005-0064-2 16228230

[B24] Schotten U. Verheule S. Kirchhof P. Goette A. (2011). Pathophysiological mechanisms of atrial fibrillation: a translational appraisal. *Physiol. Rev.* 91 265–325. 10.1152/physrev.00031.2009 21248168

[B25] Steinberg J. S. (2004). Atrial fibrillation: an emerging epidemic? *Heart (British Cardiac Society)* 90 239–240. 10.1136/hrt.2003.014720 14966028 PMC1768135

[B26] Szklarczyk D. Gable A. L. Lyon D. Junge A. Wyder S. Huerta-Cepas J. (2019). STRING v11: protein-protein association networks with increased coverage, supporting functional discovery in genome-wide experimental datasets. *Nucleic Acids Res.* 47 D607–D613. 30476243 10.1093/nar/gky1131 PMC6323986

[B27] Takeda K. Kishi H. Ma X. Yu Z. X. Adelstein R. S. (2003). Ablation and mutation of nonmuscle myosin heavy chain II-B results in a defect in cardiac myocyte cytokinesis. *Circ. Res.* 93 330–337. 10.1161/01.res.0000089256.00309.cb 12893741

[B28] Vergara P. Della Bella P. (2014). Management of atrial fibrillation. *F1000prime Rep.* 6:22. 10.12703/P6-22 PMC3974569 24765527

[B29] Wakili R. Voigt N. Kaab S. Dobrev D. Nattel S. (2011). Recent advances in the molecular pathophysiology of atrial fibrillation. *J. Clin. Invest.* 121 2955–2968. 10.1172/jci46315 21804195 PMC3148739

[B30] Waller A. P. George M. Kalyanasundaram A. Kang C. Periasamy M. Hu K. (2013). GLUT12 functions as a basal and insulin-independent glucose transporter in the heart. *Biochimica et Biophysicaacta* 1832 121–127. 10.1016/j.bbadis.2012.09.013 23041416

[B31] Wisniewski J. R. Zougman A. Nagaraj N. Mann M. (2009). Universal sample preparation method for proteome analysis. *Nat. Methods* 6 359–362. 10.1038/nmeth.1322 19377485

[B32] Xu X. Zhang P. Kwak D. Fassett J. Yue W. Atzler D. (2017). Cardiomyocyte dimethylarginine dimethylaminohydrolase-1 (DDAH1) plays an important role in attenuating ventricular hypertrophy and dysfunction. *Basic Res. Cardiol.* 112:55. 10.1007/s00395-017-0644-z PMC6502637 28819685

[B33] Yang W. Wei X. Su X. Shen Y. Jin W. Fang Y. (2019). Depletion of beta3-adrenergic receptor induces left ventricular diastolic dysfunction via potential regulation of energy metabolism and cardiac contraction. *Gene* 697 1–10. 10.1016/j.gene.2019.02.038 30790654

[B34] Ye J. Beetz N. O’Keeffe S. Tapia J. C. Macpherson L. Chen W. V. (2015). hnRNP U protein is required for normal pre-mRNA splicing and postnatal heart development and function. *Proc. Natl. Acad. Sci. U S A.* 112 E3020–E3029. 26039991 10.1073/pnas.1508461112 PMC4466706

[B35] Zhang Y. Liu Y. Zhu X. H. Zhang X. D. Jiang D. S. Bian Z. Y. (2014). Dickkopf-3 attenuates pressure overload-induced cardiac remodelling. *Cardiovasc. Res.* 102 35–45. 10.1093/cvr/cvu004 24413772 PMC6279202

[B36] Zhou Z. Y. Wan L. L. Yang Q. J. Han Y. L. Li D. Lu J. (2016). Nilotinib reverses ABCB1/P-glycoprotein-mediated multidrug resistance but increases cardiotoxicity of doxorubicin in a MDR xenograft model. *Toxicol. Lett.* 259 124–132. 10.1016/j.toxlet.2016.07.710 27491883

